# Urea-Mediated Biomineralization and Adsorption of Heavy-Metal Ions in Solution by the Urease-Producing Bacteria C7-12

**DOI:** 10.3390/microorganisms14010171

**Published:** 2026-01-13

**Authors:** Qian Yang, Xiaoyi Li, Junyi Cao, Siteng He, Chengzhong He, Chunlin Tu, Keyu Zhou, Xinran Liang, Fangdong Zhan

**Affiliations:** 1 College of Resources and Environment, Yunnan Agricultural University, Kunming 650201, China; qianyang1204@163.com (Q.Y.); 2023015@ynau.edu.cn (X.L.); fkcaojunyi@outlook.com (J.C.); sitenghe@ynau.edu.cn (S.H.); zhoukeyuzky@126.com (K.Z.); 2 China Geological Survey Kunming General Survey of Natural Resources Center, Kunming 650111, China; hechengzhong@mail.cgs.gov.cn (C.H.); tuchunlin@mail.cgs.gov.cn (C.T.)

**Keywords:** urease-producing bacteria, biomineralization, adsorption, heavy metals

## Abstract

Urease-producing bacteria (UPB) have great potential for the bioremediation of heavy-metal pollution through biomineralization and adsorption. In this study, a strain of UPB, C7-12, was isolated from heavy-metal-contaminated soil in a lead–zinc mining area and identified as *Serratia marcescens*. The heavy-metal removal ability, influencing factors, and precipitation mode of this UPB strain in solution were investigated. The cadmium (Cd) removal rate in a Cd (1 mg/L) solution from C7-12 reached 85%, and pH was the main influencing factor. With urea mediation, *S. marcescens* C7-12 biomineralizes the Cd^2+^ in solution to form CdCO_3_ and removes it through extracellular precipitation and surface adsorption. Furthermore, the removal rates of Cd^2+^, Pb^2+^, Zn^2+^ and Cu^2+^ in solution by *S. marcescens* C7-12 were 33–65%, 28–32%, 22–49%, and 38–44%, respectively. The precipitation mode involves coprecipitation of multiple heavy metals to form a mineral. These heavy metals are adsorbed on the surface of bacteria through the participation of carboxyl, amino, and phosphate functional groups and extracellular polymeric substances. Therefore, *S. marcescens* C7-12 has strong biomineralization and adsorption capacity for heavy-metal ions in solution, which can provide potential resources for the bioremediation of heavy-metal-contaminated soil and water.

## 1. Introduction

Heavy-metal pollution is a global environmental problem. The mining industry is a major source of heavy-metal pollution [1]. Cd, an associated element in ores, is one of the most biotoxic heavy metals and is often released during metal smelting, harming the health of the ecosystem [2,3]. Remediation technologies, such as chemical precipitation, ion exchange, and membrane adsorption treatment, require exogenous interference to drive the directional movement of Cd^2+^ or initiate a chemical reaction for its removal. However, this process may disrupt the internal balance of the medium, produce secondary pollution, and increase remediation costs [4]. As ‘purifiers’ of environmental pollutants, microorganisms can convert soluble Cd in the environment into a more stable form through biological processes, such as catabolism and complexation [5]. This self-defense mechanism, which arises in response to stress, offers a new strategy for the remediation of Cd contamination.

UPB are widely present in Cd-contaminated soil, such as *Burkholderia* sp. [6] Bacillus cereus [7]. Wang et al. [8] screened a urease-producing bacterium TJ6 from Cd-contaminated farmland, which can significantly reduce the Cd concentration in the solution. Han et al. [9] reported a *Bacillus megaterium* significantly reduced the condition of Cd^2+^ in soil solution (Cd 1.28 mg/kg). UPB comprise a class of microorganisms with a high-efficiency urease production capacity [10]. UPB can utilize biomineralization to mineralize Cd from an ionic state to a stable state [11], specifically by secreting urease to produce CO_3_^2−^, which is subsequently deposited with Cd^2+^ to form carbonate precipitates. This process achieves the immobilization of heavy metals, thereby reducing the mobility and toxicity of harmful metals [12,13]. In this process, heavy-metal ions with similar ionic radii (e.g., Cd^2+^, Pb^2+^, Zn^2+^, and Cu^2+^) enter the mineral lattice via substitution or mosaicking [14]. Bioadsorption uses extracellular polymeric substances (EPS) secreted by cells and negatively charged groups on the surface of the cell to attract positively charged heavy-metal cations [15]. On this basis, bacteria can also act as crystallographic sites. UPB continue to attract heavy-metal ions and form minerals on their surfaces. These mineral particles are superimposed repeatedly until the bacteria are completely encapsulated, resulting in larger particle sizes and more stable mineral precipitation [16,17].

In addition, the ability of UPB to remove Cd is easily controlled and affected by environmental factors. For example, urea concentration, as a key substrate in the biomineralization process, significantly impacts the reaction rate: low concentrations constrain the rate, while high concentrations may inhibit bacterial growth, thereby reducing urease activity [18]. Similarly, an initial heavy-metal concentration that exceeds bacterial tolerance limits reduces the removal efficiency of heavy-metal ions [19]. Furthermore, the inoculum size [20], temperature [21], and pH [22] also govern the Cd removal capacity of urease-producing bacteria. Consequently, the effectiveness of heavy-metal immobilization under varying environmental conditions is a crucial indicator for assessing the practical applicability of bacterial strains.

Heavy-metal pollution is a composite of multiple heavy metals, which presents challenges for the removal of heavy-metal ions by UPB. However, the effects of urea on the biomineralization process and the site of action of UPB and the removal effects and precipitation modes of UPB on heavy-metal ions in contaminated soil solutions remain ambiguous. In this study, the effects of environmental factors (temperature, pH, urea concentration, bacterial inoculation amount, incubation time and initial Cd concentration) on the removal of Cd were investigated by screening for Cd urease-resistant bacteria. Moreover, the removal mechanism of Cd by UPB under different urea concentrations was clarified. To further explore the removal effects and precipitation patterns of UPB on multiple heavy metals. The following hypotheses are proposed: (1) Urea-mediated biomineralization occurs in the extracellular environment, and the removal of Cd is based mainly on the formation of CdCO_3_ induced by bacterial activity. (2) UPB has the ability to remove multiple heavy-metal ions simultaneously and reduce the concentration of heavy=metal ions in solution to form multiple heavy-metal mixed minerals.

## 2. Materials and Methods

### 2.1. Bacterial Strains, Culture Media and Contaminated Soil Solutions

The test strains were selected from 65 UPB, obtained from the rhizosphere soil of maize plants planted around a lead–zinc mining area. All the strains were kept in beef paste peptone agar slants at 4 °C. To maintain their viability, the cells were subcultured at 2-month intervals [23].

The medium used in this experiment was Luria broth (LB) liquid medium (pH = 7.0–7.3), which consisted of yeast extract (5 g/L), peptone (10 g/L), and NaCl (5 g/L) [24]. The solid LB medium was prepared by adding 20 g/L agar to the liquid medium and sterilizing it at 121 °C for 20 min. Urea (30 g/L) was filter sterilized via a 0.45 μm filter and added to the cooled LB medium.

The soil solution used in this experiment was obtained from slag (K, 26.3052° N, 103.3572° E), contaminated soil (W, 26.3047° N, 103.3579° E) and farmland soil (N, 26.3039° N, 103.3592° E) from a smelter in Huize, Yunnan Province. The basic physical and chemical indices of the soil are shown in Table S1. The soil samples were added to pure water at a ratio of 1:1 (*m*/*m*) for 12 h and then filtered through a 0.22 μm filter membrane [9]. The physical and chemical properties and the anion and cation contents of the soil solutions are shown in Table 1.

### 2.2. Screening of Cd Urease-Resistant Bacteria

To investigate the ability of strain C7-12 to resist Cd, the half-maximal effective concentration (EC_50_) of Cd was used as the criterion for determining Cd resistance. LB medium (100 mL) containing Cd solutions of different concentrations (0, 20, 40, 80, 160, and 320 mg/L) was inoculated separately with 2% inoculum of UPB (OD_600_ = 0.4; the total amount of bacteria was 7.6 × 10^7^ CFU/mL). The experiments were carried out at 30 °C with shaking at 160 rpm. Based on the EC_50_ values, Cd-resistant bacteria were selected. Each experiment was replicated three times, and the specific test process is shown in Figure S1.

### 2.3. Identification of UPB

The bacterial barcoding gene 16S rDNA was identified via the universal primers 27F (5′-AGAGTTTGATCCTGGCTCAG-3′) and 1492R (5′-CTACGGCTACCTTGTTACGA-3′). Strain identification was conducted via 16S rDNA sequencing, and similar DNA sequences were matched in the NCBI database via BLAST (https://blast.ncbi.nlm.nih.gov/Blast.cgi, accessed on 28 July 2025). Phylogenetic trees were constructed via the neighbor‒joining method of MEGA5.1 [25], and confidence testing was conducted through bootstrapping (1000 resamplings) to determine the position of the tested strain species in the classification system [26].

### 2.4. Urease Activity and Urea Concentration

The urease activity in the supernatant was determined according to the methods of Qin et al. [27]. 0.1 mL of the supernatant was added to 0.9 mL of a urea solution (3%). The mixture was incubated at 35 °C for 7 min. Afterward, the reaction was stopped by adding 1 mL of trichloroacetic acid solution (10%). After the sample was cooled, 1 mL of Nessler’s reagent was added and diluted to 25 mL, and after 20 min of color development, the OD415 was determined. One unit of urease is defined as the amount of 1 mmol of NH_4_^+^ per minute. The formula for calculating the urease activity of UPB was as follows:

(1) U = M/(0.1 × 7) × 250

where M is the amount of NH_4_^+^ in the sample.

Urea concentration in the supernatant: 14 mL of the 100-fold diluted supernatant was added to 10 mL of Ehrlich’s reagent. After 30 °C for 10 min of color development, the OD_426_ was determined. The formula for calculating the urea consumption in grams per liter (g/L) can be used to quantify the amount of urea consumed during the process:

(2) urea consumption (g/L) = initial urea concentration (g/L) − residual urea concentration (g/L)

### 2.5. Cd Removal Experiment

LB medium (100 mL) containing 30 g/L urea as an initial cultural condition, with different concentrations of Cd^2+^ (5, 10, 20, and 40 mg/L) was inoculated separately with 2% inoculum of Cd-resistant UPB and cultured at 30 °C and 160 rpm for 48 h. The samples were subsequently centrifuged at 8000 rpm and 4 °C for 10 min, after which the supernatants were collected. The Cd^2+^ concentration in the supernatant was determined via a flame atomic spectrophotometer. Through differential analysis, the urease-producing bacteria with the strongest Cd removal ability were ultimately screened. The formula for calculating the Cd^2+^ removal rate of UPB was as follows [28]:

(3) removal rate (%) = (C_0_ − C_s_)/C_0_ × 100%

where C_0_ and C_s_ are the Cd concentration in the supernatant before bacterial treatment and that after bacterial treatment, respectively.

### 2.6. Effects of Environmental Factors on Cd Removal by UPB

The one factor per time (OFT) method was used to explore the influence of environmental factors on the removal of Cd by UPB and optimize the medium conditions. The environmental factors used were as follows: initial Cd concentration (mg/L): 0.1, 1, and 5; incubation time (h): 0, 4, 8, 12, 24, 36, 48, 72, and 96; temperature (°C): 20, 25, 30, and 35; bacterial inoculation amount (%): 1, 2, 4, and 8; and urea concentration (g/L): 0, 10, 20, 30, 40, and 50. Each experiment was replicated three times.

### 2.7. Determination of Heavy-Metal Concentrations in Bacterial Extracellular Precipitation, Surface Adsorption, and Intracellular Accumulation

Soil solution was added to the LB medium. Under optimized cultural conditions, the medium was inoculated with a 2% inoculum of C7-12. After 48 h of cultivation, the surface-adsorbed heavy metals were determined, and the measurement method referred to Han et al. [29]. Strain C7-12 was incubated in the optimal medium for 48 h. The bacterial pellets and supernatant were collected by centrifugation at 8000 rpm for 10 min. The bacterial precipitate was washed with 0.02 mol/L ETDA-2Na washing solution 3 times, and the washing solution was combined to determine the heavy-metal content, which was the surface heavy-metal adsorption content. A total of 15 mL of digestive juice (perchloric acid:hydrogen peroxide:concentrated sulfuric acid = 3:3:1) was added to the bacterial pellet after washing, and the heavy-metal content was determined via high-temperature digestion to calculate the intracellular heavy-metal accumulation. To avoid errors in heavy-metal adsorption due to the inner wall of the triangular flask, the inner wall was rinsed three times with nitric acid. The washing solution was then collected, and its heavy-metal content was determined. The residual heavy-metal ions on the inner wall of the triangular flask were calculated, and the specific test process is shown in Figure S2. The amount of extracellular heavy-metal precipitation was calculated as follows:

(4) amount of extracellular heavy-metal precipitation = A − B − C − D − E

where A represents the initial heavy-metal content; B represents the adsorption capacity of the triangular bottle for heavy metals on the inner wall; C represents the adsorption capacity of heavy metals on the surface; D represents the amount of accumulated heavy metals; and E represents the heavy-metal content of the supernatant.

### 2.8. Bacterial Characterization Analysis

The surface morphology of the bacterium and the surface elemental composition were imaged and determined by electron microscopy and energy dispersive spectrometry (SEM‒EDS, Hitachi SU3800 Hitachi MC1000, Tokyo, Japan). The samples were analyzed on a Fourier transform infrared spectrometer (FTIR, Thermo Scientific iN10, Waltham, MA, USA) to evaluate the changes in functional groups on the surface of the bacterial cells. X-ray diffraction (XRD, Rigaku Ultima IV, Tokyo, Japan) was carried out to measure the crystalline phases of the bacterial surface precipitates.

### 2.9. Data Analysis

The means and standard deviations (SDs) of all the data were calculated via Excel 2021 and plotted via Origin 2021 software. SPSS 27 was used for difference analysis, and the statistical significance was *p* < 0.05. The sequencing results of the isolated strains were used to construct a phylogenetic tree with MEGA 5.1.

## 3. Results

### 3.1. Screening and Identification of UPB

**Figure 1:**
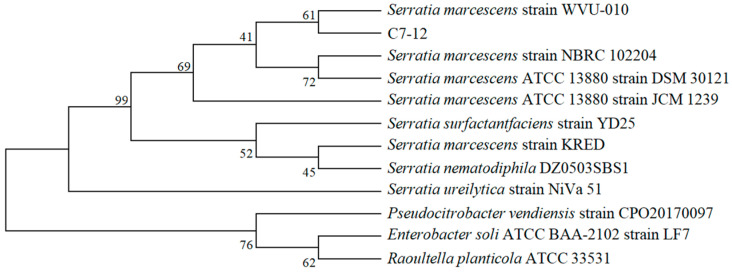
Phylogenetic tree of strain C7-12 based on 16S rDNA gene.

After the Cd resistance experiment (0~320 mg/L Cd) (Figure S3) and the Cd removal experiment (5~40 mg/L Cd) (Figure S4) were conducted, strain C7-12 was isolated. This strain showed high Cd resistance (Figure S5) and significant Cd removal ability in solution. This strain was subsequently selected for further research. A comparison of the 16S rDNA sequence and NCBI database BLAST data revealed that the nearest bacterial species to the C7-12 isolate was *Serratia marcescens*, with 100% matching (Figure 1). Currently, strain C7-12 (GDMCC65568) is preserved in the Guangdong Microbial Culture Collection Center (GDMCC).

### 3.2. Effects of Environmental Factors on the Removal of Cd from Solution by UPB

#### 3.2.1. Initial Cd Concentration and Time

**Figure 2:**
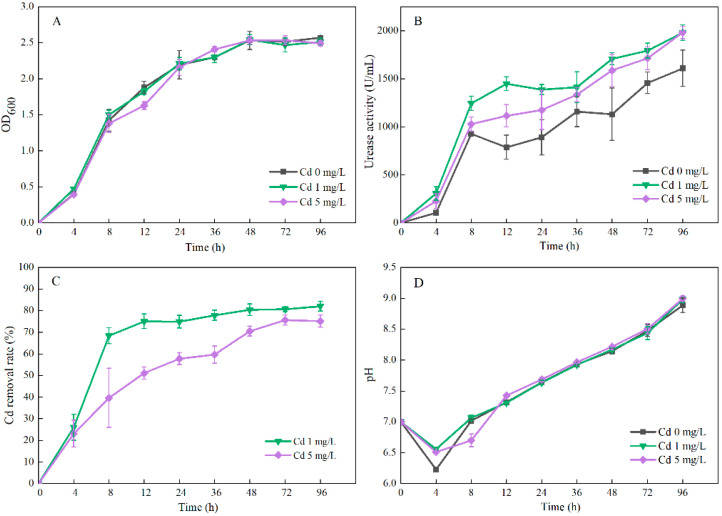
Effects of initial Cd concentration and incubation time on OD_600_ (**A**), urease activity (**B**), Cd removal rate (**C**) and solution pH (**D**) of UPB.

Compared with the treatment without Cd, strain C7-12 grew normally (Figure 2A) and presented increased urease activity (Figure 2B) at 1 and 5 mg/L Cd. With increasing Cd concentration, the removal rate of bacterial pairs decreased. Compared with 5 mg/L Cd, strain C7-12 presented a greater removal rate of 1 mg/L Cd (Figure 2C). After 48 h of incubation, the Cd removal rate was essentially stable, and the pH of the solution was greater than 8.3 (Figure 2D). In summary, the removal rate was the highest at an initial Cd concentration of 1 mg/L, and the incubation time was 96 h, which was 82%. To save time and cost, the incubation time was 48 h, and the initial Cd concentration was 1 mg/L.

#### 3.2.2. Bacterial Inoculation Amount, Urea, Temperature and pH

**Figure 3:**
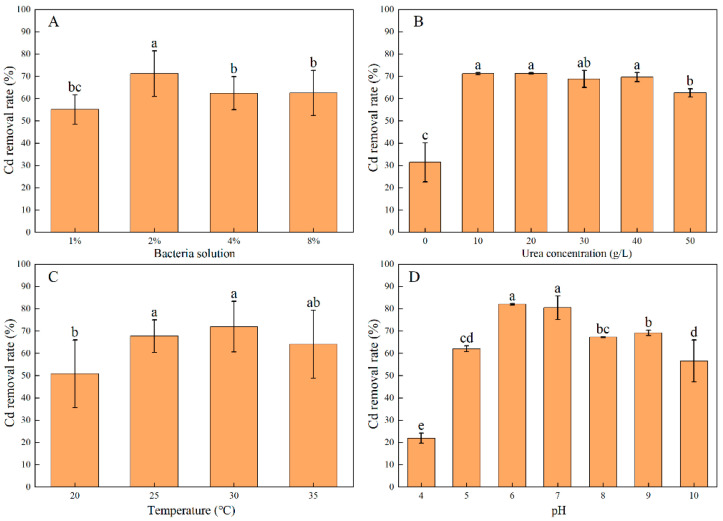
Effect of inoculation amount (**A**), urea concentration (**B**), temperature (**C**) and pH (**D**) on the Cd^2+^ removal rate of UPB. Different lowercases in the same chapter represented a significant difference (*p* < 0.05) between different dosages.

The Cd removal rate of strain C7-12 tended to increase but then decreased as the bacterial inoculation amount increased. The Cd removal rate peaked at the 2% inoculation amount, at which value it reached 71% (Figure 3A). Hence, the inoculation amount of 2% was used in subsequent experiments.

Under 10–40 g/L urea, strain C7-12 presented no significant difference in terms of the Cd removal rate. Under 20 g/L urea, C7-12 presented the highest Cd removal rate (71%) (Figure 3B). Hence, a urea concentration of 20 g/L was used in subsequent experiments.

The Cd removal rate of strain C7-12 tended to increase but then decreased as the temperature increased. At 25–35 °C, the Cd removal rate of strain C7-12 did not significantly differ. At 30 °C, C7-12 presented the highest Cd removal rate (79%) (Figure 3C). Therefore, a temperature of 30 °C was used in subsequent experiments.

Strain C7-12 presented the lowest Cd removal rate (22%) at pH = 4 and the highest Cd removal rate (82%) at pH = 6. When the pH was >7, the Cd removal rate of strain C7-12 tended to decrease (Figure 3D). In summary, pH had the greatest influence on the Cd removal rate within the range of environmental factors, with a difference of up to 60%.

The medium culture conditions for strain C7-12 are shown in Table 2. The Cd removal rate of C7-12 reached 85%. The OD_600_, urease activity, and pH of strain C7-12 under optimal medium conditions were 1.10, 1.23 and 1.04 times greater than those of the original basal medium, respectively. These optimized culture conditions increased the growth of C7-12 and increased both urease activity and alkali production capacity (Table 2).

### 3.3. Biomineralization of UPB Under the Action of Urea

#### 3.3.1. Cd Distribution and Urea Consumption of UPB at Different Urea Concentrations

**Figure 4:**
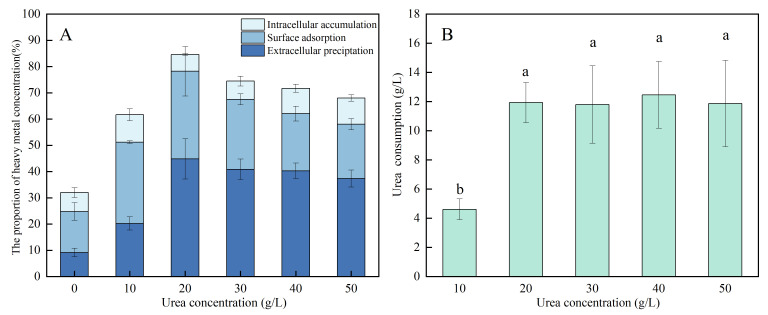
Distribution of Cd in different parts of bacteria (**A**), urea consumption (**B**) under different urea concentrations. Different lowercases in the same chapter represented a significant difference (*p* < 0.05) between different dosages.

Under 20 g/L urea, strain C7-12 achieved the highest degree of Cd^2+^ extracellular precipitation (about 45%) and surface adsorption (about 33%) and the lowest degree of intracellular accumulation (about 6%). At a urea concentration of 0 g/L, strain C7-12 achieved the lowest degree of Cd^2+^ extracellular precipitation (about 6%) and surface adsorption (about 16%) (Figure 4A). Urea consumption increased with increasing urea concentration. Strain C7-12 did not significantly differ in terms of urea consumption (11.8–12.5 g/L) in media supplemented with 20–50 g/L urea (Figure 4B).

#### 3.3.2. Correlation Analysis of Extracellular Precipitation, Surface Adsorption, and Intracellular Accumulation of Cd and Urea Consumption in UPB

**Figure 5:**
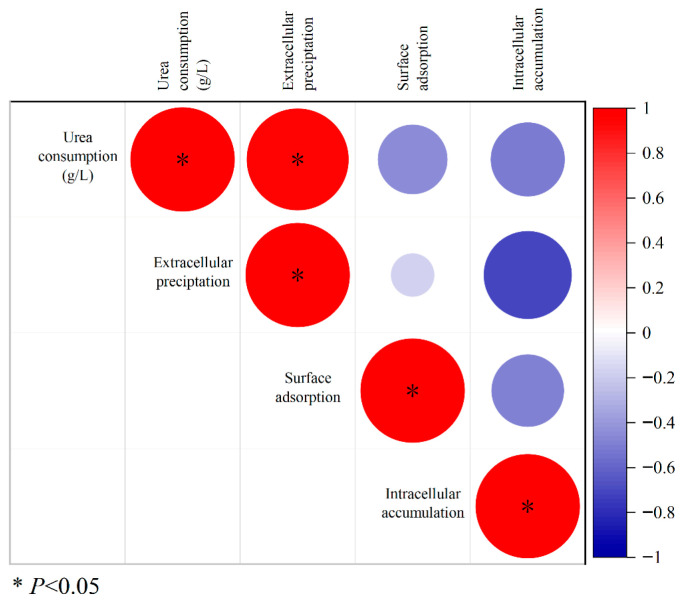
Correlation analysis between Cd concentration and urea consumption in each part of strain C7-12.

Correlation analysis revealed a significant positive correlation between the concentration of Cd in the extracellular precipitate of C7-12 and urea consumption. This finding indicates that the biological mineralization influenced by urea occurs mainly outside the cell, fixing Cd^2+^ through precipitation (Figure 5).

#### 3.3.3. SEM-EDS Analysis

**Figure 6:**
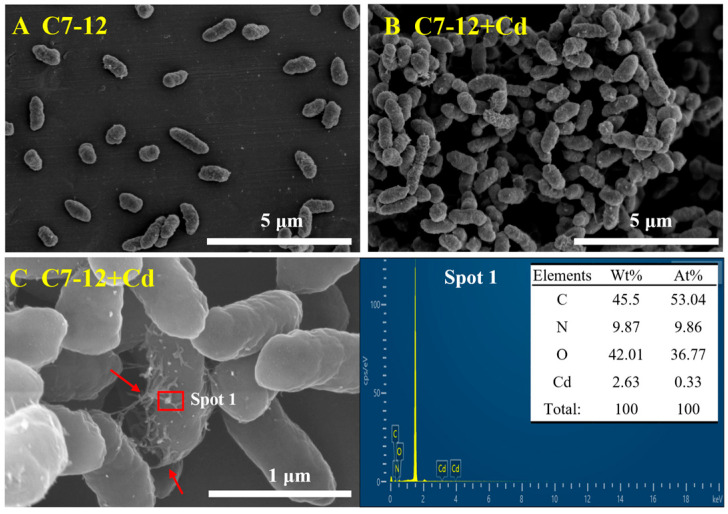
SEM and EDS images before and after Cd treatment. Panels (**A**,**B**) show SEM images. Panel (**C**) show the EDS scanning area and mineral elements on cell surface (The arrows indicate the EPS).

SEM revealed that strain C7-12 exhibited a full morphology, a short rod-shaped form, and was sparsely distributed at 0 mg/L Cd (Figure 6A). Under 1 mg/L Cd, the bacteria formed agglomerates, and extracellular polymers and particulate matter appeared on the surfaces of the strains (Figure 6B,C). The morphology of the strains remained largely unchanged compared with that of the strains prior to the adsorption treatment. EDS analysis revealed that the surfaces of the precipitates formed by C7-12 were carbon (C), nitrogen (N), oxygen (O), and Cd. The C7-12 strain exhibits the capacity to adsorb Cd^2+^ ions and facilitate their precipitation on its cell surface, and the observed reduction in aqueous Cd^2+^ concentrations is likely attributable to the microbially induced biomineralization process mediated by this strain.

#### 3.3.4. FTIR and XRD Analysis

**Figure 7:**
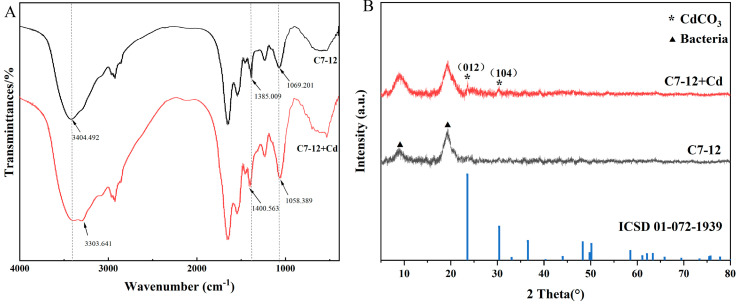
FTIR (**A**) and XRD (**B**) images of bacteria before and after Cd treatment.

The FTIR absorption peak of strain C7-12 after Cd adsorption changed in position compared with that before adsorption. The characteristic peaks of -OH [30] and C-N [31] shifted from 1069 cm^−1^ to 1058 cm^−1^. The characteristic CO_3_^2−^ [32] peak shifted from 1385 cm^−1^ to 1401 cm^−1^, and the characteristic peak of -OH shifted from 3404 cm^−1^ to 3304 cm^−1^, indicating that these functional groups may be involved in the adsorption process of Cd (Figure 7A).

The structural transformations of Cd^2+^ was tracked using XRD after 48 h of reaction. Compared with standard diffraction patterns of otavite (CdCO_3_ (ICSD No. 01-072-1939)), the XRD reflections in CdCO_3_ film are located at 23.49° and 30.28° after 48 h of reaction. Its low solubility (Ksp = 3.8 × 10^−14^) ensures that the immobilized Cd can remain stable for a long time in the natural environment. The primary substance adsorbed on the surface of strain C7-12 is most likely otavite (Figure 7B).

### 3.4. UPB for the Removal of Heavy-Metal Ions in Solution

**Figure 8:**
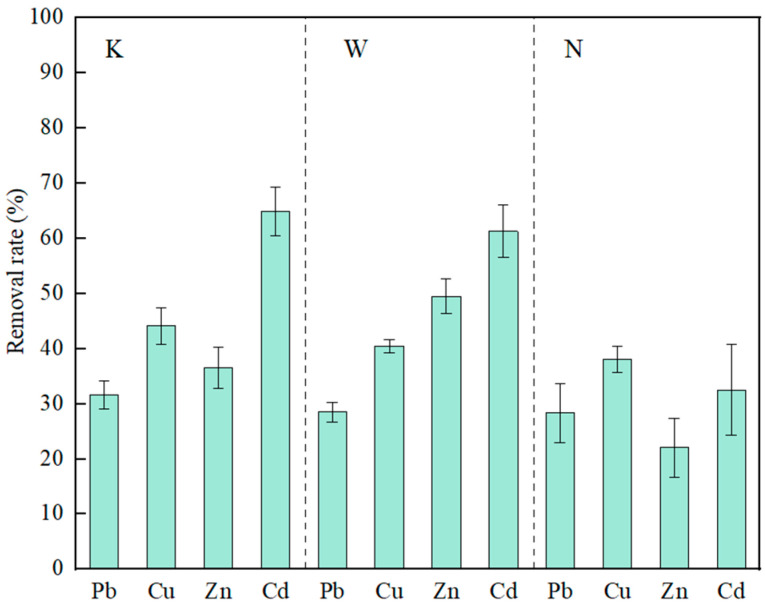
Removal of the four heavy-metal ions by strain C7-12.

In the slag solution (K), strain C7-12 had the highest removal rate of Cd (65%) and the lowest removal rate of Pb (32%). In the contaminated soil solution (W), the removal rate of Cd was the highest, at 61%, and the removal rate of Pb was the lowest, at 28%. In the farmland soil solution (N), the removal rate of Cu was the highest, at 38%, and the removal rate of Zn was the lowest, at 22% (Figure 8).

### 3.5. Mechanism of Heavy-Metal Ion Removal in Solution by UPB

#### 3.5.1. Distribution of Heavy-Metal Ions in Various Parts of UPB

**Figure 9:**
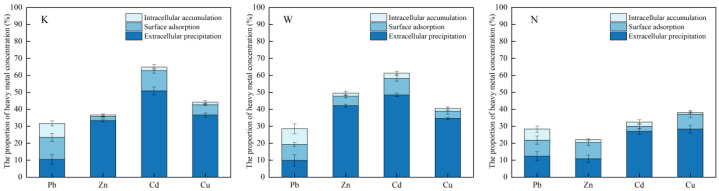
Distribution of heavy-metal ions in each part of bacteria.

The extracellular precipitation of strain C7-12 plays a more significant role in the removal process of heavy-metal ions. In both the slag (K) and contaminated (W) soil-solution treatments, zinc (Zn) removed via extracellular precipitation by strain C7-12 accounted for the highest percentage of total Zn removal, at around 91% and 85%, respectively. In the farmland (K) soil solutions, the Cd removed via extracellular precipitation accounted for the highest percentage of total Cd removal, at around 83% (Figure 9).

#### 3.5.2. SEM-EDS and XRD Analysis

**Figure 10:**
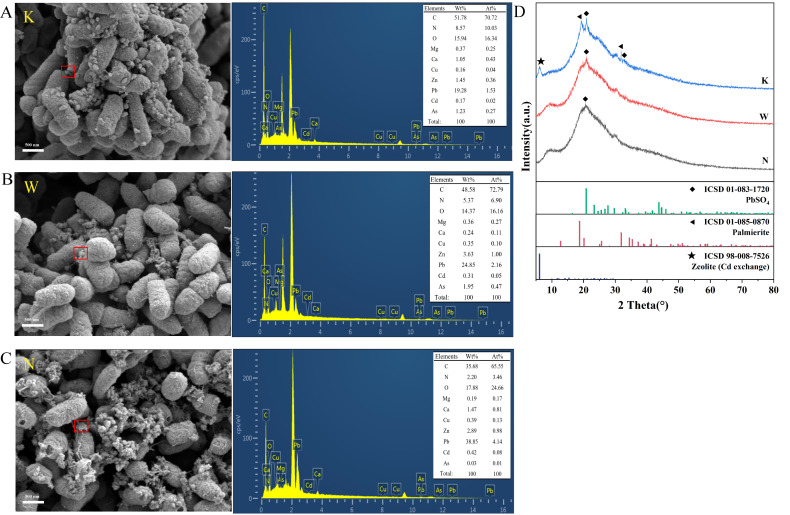
Images of SEM-EDS (**A**–**C**) and XRD (**D**) of strain C7-12 under soil-solution treatment. The red squares marks the EDS scanning area.

SEM revealed that the morphology of the cells did not change significantly and that the surfaces of the bacteria were tightly coated with EPS and particulate precipitates. EDS analysis revealed the presence of multiple heavy-metal signals in the surface-adsorbed precipitates. The percentages of Pb and Zn are the largest. Therefore, we can preliminarily suggest that the heavy-metal resistance and adsorption capacity of C7-12 are related to the secretion of EPS (Figure 10A–C). PbSO_4_ (ICSD 01-083-1720), palmierite (ICSD 01-085-0870), and zeolite (Cd exchange) (ICSD 98-008-7526) were detected on the bacterial surface in the K soil-solution treatment. In the W and N soil-solution treatments, PbSO_4_ (ICSD 01-083-1720) may be present on the bacterial surface (Figure 10D). In summary, the formation of minerals on the bacterial surface is related to the concentration of heavy-metal ions in the environment.

#### 3.5.3. FTIR Analysis

**Figure 11:**
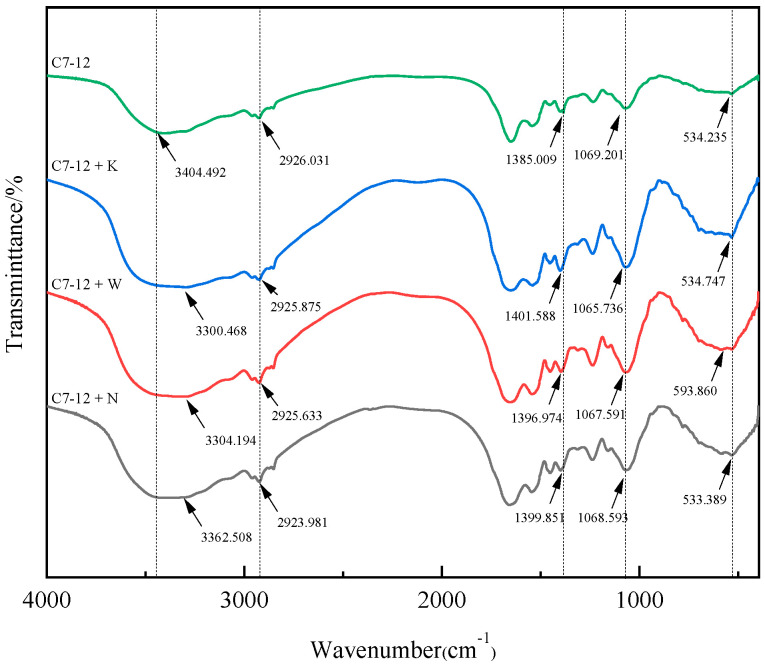
FTIR image of strain C7-12 under soil-solution treatment.

The FTIR results revealed that the positions of the absorption peaks representing amino, carboxyl, phosphoric and sulfuric acid groups shifted after soil-solution treatment. The characteristic -OH peak of the polysaccharide shifted from 3400 cm^−1^ to 3300–3363 cm^−1^, with an additional shift observed at 2930 cm^−1^. The characteristic CO_3_^2−^ peak of polysaccharides shifted from 1385 cm^−1^ to 1397–1402 cm^−1^. The characteristic phosphoric acid and sulfuric acid group peaks of the polysaccharides shifted from 534 cm^−1^ to 533–594 cm^−1^, indicating that both EPS (primarily composed of polysaccharides) and functional groups on the bacterial surface were involved in the adsorption of heavy metals (Figure 11).

## 4. Discussion

### 4.1. Effects of Different Environmental Factors on the Response of UPB to Cd Removal

As a living organism, the removal effect of UPB on Cd is affected by a variety of environmental factors [33,34]. We investigated the effects of the initial Cd concentration, incubation time, temperature, urea, inoculation amount and pH on the Cd^2+^ removal ability of UPB. We discovered that the pH had the greatest effect on the removal of Cd^2+^. The Cd removal rate was lowest at pH = 4, which was 60% different from the Cd removal rate at pH = 6. *Bacillus cereus* screened by EL-Meihy et al. is more suited to survival in neutral environments, which demonstrates that strain C7-12 has adaptability to acidic environments and thus exhibits broader environmental adaptability [22]. This indicates that the growth capacity of UPB in a strongly acidic environment (pH = 4) is impaired, inhibiting urease synthesis and secretion. Meanwhile, strongly acidic and alkaline conditions induce conformational changes in urease proteins, hindering urea hydrolysis. When the pH < 7, H^+^ competes with heavy-metal ions for binding sites, resulting in a decrease in the adsorption efficiency. Moreover, Cd^2+^ is reactivated from mineral precipitation due to electrical repulsion [35]. Furthermore, the Cd^2+^ removal rate by UPB is also significantly influenced by urea concentration. The urea concentration had the lowest Cd removal rate of 31% in the urea-free treatment, which was 40% different from the optimal urea-concentration treatment. As the pH increases, the urease activity of UPB increases, which promotes the hydrolysis of urea, accelerates the conversion of HCO_3_^−^ to CO_3_^2−^, and attracts more Cd^2+^. At low urea concentrations, urease captures less urea per unit time, resulting in low CO_3_^2−^ concentrations, which are not conducive to carbonate precipitation. At high urea concentrations, the growth of UPBs can be inhibited. A high urea concentration can destroy the EPS structure, resulting in a decrease in the Cd removal rate [36]. Urea hydrolysis is essential for pH elevation and CO_3_^2−^ provision, with its efficacy governed by UPB-derived urease secretion and activity.

This study adopted the OFT method to investigate the effects of various environmental parameters on the Cd^2+^ removal efficiency of UPB. This method can intuitively screen out the key parameters that have a significant impact on Cd removal efficiency. In the future, multi-variable or factorial experimental methods (such as response surface methodology, orthogonal experimental design, etc.) can be used to fully consider the possible interactions between various environmental parameters. Strain C7-12 exhibited excellent heavy-metal ion removal capacity in soil solutions with different pollution levels. Future studies should apply it to the remediation of actual heavy-metal-contaminated soils to verify its environmental applicability.

### 4.2. Study of the Mechanism of Cd Removal by UPB Under the Action of Urea

The process of biomineralization involving urea hydrolysis mediated by UPB has been demonstrated to be an effective strategy for reducing the Cd^2+^ concentration [37]. In this study, we specifically focused on and quantitatively revealed a direct correlation between the concentration of extracellular Cd precipitation and urea consumption. With increasing urea concentration, the extracellular Cd^2+^ precipitation by UPB initially increased and then stabilized. At urea concentrations of 20–40 g/L, the precipitated Cd^2+^ was significantly higher than that in other treatment (0 and 10 g/L urea). Correlation analysis demonstrated that the extracellular Cd^2+^ concentration was significantly correlated with urea consumption. This indicates that, under appropriate urea concentration conditions, biomineralization driven by urea hydrolysis is the dominant mechanism for immobilizing Cd^2+^ in the solution. This study also revealed that the Cd precipitation formed by C7-12 contained CdCO_3_. It is a key evidence mineral for Cd^2+^ to be fixed by CO_3_^2−^, marking the transition of Cd from a migratory ionic state to a stable solid state. Owing to the low concentration of Cd in this experiment, the precipitate generated exhibited a small particle size, and it was encapsulated by EPS, ultimately resulting in a significantly lower Cd content than those of C, N, and O [38]. We plan to quantify the content of EPS in the system and further explore the correlation between EPS content and Cd adsorption capacity.

This study revealed that the intracellular accumulation of Cd^2+^ in strain C7-12 was between 6% and 11%. Cd^2+^ enters the cell through metal protein channels located on the cell wall. With the assistance of ABC transporter proteins, Cd^2+^ is transported to specific compartments, such as vacuoles, for immobilization. Ultimately, the toxicity of Cd is reduced through efflux or accumulation mechanisms [39]. However, dead bacteria also have the ability to remove Cd, but their removal capacity is lower than that of live bacteria. The main difference is that dead bacteria cannot carry out intracellular Cd accumulation [40]. Therefore, it will be necessary to quantify the survival rate of bacteria in the system to better demonstrate the contribution of live bacteria’s biomineralization ability to Cd removal. The leaching method employed in this study is essentially a semi-quantitative analytical approach. The selectivity of different extractants is not absolute; some bound Cd may undergo speciation transformation due to fluctuations in extraction conditions, leading to certain errors in the quantitative results of each fraction. Therefore, the Cd distribution ratio of strain C7-12 exhibits a relative trend of extracellular precipitation > surface adsorption > intracellular accumulation.

### 4.3. Study of the Removal Mechanism of UPB from a Variety of Heavy-Metal Ions in Solution

We found that, compared with the single Cd treatment, the displacement of surface functional groups occurred at consistent characteristic peak positions. Additionally, the contribution of surface adsorption to the removal of heavy-metal ions is reduced under soil-solution treatment. We conclude that bacterial surface functional groups have the capacity to adsorb a significant proportion of heavy metals, and there is no specificity for the adsorption of heavy metals by functional groups on the surface of bacteria.

EPSs are composed mainly of polysaccharides and glycoproteins, which can provide more Cd^2+^ adsorption sites in the bacterial cell wall. This greatly improves the adsorption efficiency of Cd^2+^ [41,42,43]. UPB produces EPS, which can wrap around the bacterial body to resist the adverse effects of the environment on bacteria [44,45]. Sharma and Shukla [46] observed the presence of EPS around bacteria via SEM; our study revealed the same results. In this experiment, the surface of the cell was tightly wrapped by the secreted extracellular polymer, the silk-like extracellular polymer was connected with the cells, and the precipitate on the surface of the cell was coated with EPS. This phenomenon is the response of UPB to the adverse effects of heavy-metal toxicity [47,48]. Mathivanan et al. [49] reported that the yield of EPS increased from 1099 μg/mL to 1185.5 μg/mL under Bacillus cereus KMS3-1 stress at a concentration of 50 mg/L Cd^2+^. Owing to the larger surface area of EPS for bacterial cells to interact with heavy-metal ions and the greater number of negatively charged functional groups present in the environment, capturing heavy-metal ions is easier. We observed that strain C7-12 possesses the capacity to secrete extracellular polymeric substances (EPS) and perform biomineralization, leading to the more efficient immobilization of Cd^2+^ under the synergy of adsorption and precipitation.

In this study, the precipitates attached to the surface, which exhibited a novel shape, were composed of multiple heavy metals. XRD analysis indicated that these might include minerals such as PbSO_4_, palmierite, and zeolite (Cd exchange). The potential origins of these substances include the following: (1) SEM-EDS analysis found that, in addition to Pb, Zn, Cu and Cd, elements such as Ca and Mg were also detected on the surface of the precipitates. These elements are among the most common in the local soil. In addition, palmitite is a common substance in the mining area, so its presence on the bacterial surface may originate from minerals in the water that became attached to the bacteria through biological processes, (2) minerals generated by the biomineralization of urease-producing bacteria (UPBs). The characteristic vibration peaks of sulfate groups detected by Fourier transform infrared spectroscopy (FTIR) confirmed that Pb^2+^ formed PbSO_4_ with the participation of functional groups on the bacterial surface, and the excellent biomineralization and adsorption capacities of UPBs (Section 3.3) further provided nucleation and mineralization conditions for heavy-metal precipitation, and (3) minerals formed due to pH changes from bacterial metabolic activities [50]. However, the potential interference of inherent microminerals, Ca^2+^ and Mg^2+^ in the soil solution cannot be ruled out, and this issue will be further verified in subsequent studies.

Strain C7-12 exhibits considerable potential for the remediation of heavy-metal-contaminated environments, and previous studies have demonstrated that *Serratia marcescens* can synergize with plants to enhance heavy-metal remediation efficiency [51,52]. However, *S. marcescens* is generally recognized as an opportunistic pathogen in clinical settings [53,54]. Notably, microbial pathogenicity is strongly strain-specific and host-dependent. To ensure the safety and engineering feasibility of its environmental applications, future research should prioritize small-scale field trials at industrial contaminated sites with low population density or non-potable water sources. Alternatively, strategies such as material immobilization can be adopted to prevent the strain’s environmental dissemination and alleviate the issues of nutrient limitation and activity attenuation faced by the strain in real-world scenarios. Concurrently, it is imperative to evaluate the long-term stability of precipitated minerals in natural environments, thereby comprehensively mitigating potential ecological risks.

## 5. Conclusions

Strain C7-12, which exhibited high Cd resistance and superior removal efficiency, was identified as *Serratia marcescens*. Strain C7-12 was inoculated into a culture medium containing 1 mg/L Cd^2+^, with a pH of 6 and a 20 g/L urea concentration, at 2% bacterial inoculum. After incubation at 30 °C for 48 h, an 85% removal efficiency was observed. The primary mechanisms for the removal of Cd by strain C7-12 involve extracellular precipitation and surface adsorption. Extracellular precipitation primarily relies on the hydrolysis of urea by urease to induce the formation of CdCO_3_ from Cd^2+^, whereas surface adsorption is associated with EPS and functional groups on the bacterial surface. Furthermore, strain C7-12 can drive the coprecipitation of multiple heavy-metal ions and has high potential for practical use in complex toxic metal-polluted water treatment.

## Figures and Tables

**Table 1:** Physicochemical properties and anion/cation contents of soil solutions.

Soil Solutions	Pb^2+^ (mg/L)	Zn^2+^ (mg/L)	Cd^2+^ (mg/L)	Cu^2+^ (mg/L)	Ca^2+^ (mg/L)	Mg^2+^ (mg/L)	SO_4_^2−^ (mg/L)	Cl^−^ (mg/L)	pH
K	11.47 ± 1.14	13.96 ± 0.61	2.10 ± 0.41	6.04 ± 0.69	2.35 ± 0.12	0.53 ± 0.04	7.38 ± 0.40	12.08 ± 2.54	4.57 ± 0.11
W	10.86 ± 1.17	9.15 ± 0.81	1.46 ± 0.39	3.62 ± 0.09	4.57 ± 0.4	1.26 ± 0.27	2.8 ± 0.25	8.13 ± 1.78	5.06 ± 0.02
N	7.87 ± 0.47	3.98 ± 0.06	0.10 ± 0.01	1.74 ± 0.05	6.22 ± 1.33	1.46 ± 0.07	20.5 ± 1.13	9.21 ± 1.52	5.76 ± 0.07

Note: K represents the slag solution, W denotes the contaminated soil solution, and N signifies the farmland soil solution. All subsequent instances are replaced with the corresponding letters.

**Table 2:** Cd removal rate, OD_600_, urease activity, and pH change in culture under optimal conditions.

	Index	Value
Conditions	Initial Cd concentration (mg/L)	1
	Time (h)	48
	Inoculation amount (%)	2
	Urea concentration (g/L)	20
	Temperature (°C)	30
	pH	6
C7-12	Cd removal rate (%)	84.61 ± 5.81
	OD_600_	2.84 ± 0.12
	Urease activity (U/mL)	2107.7 ± 184.0
	ΔpH	2.51 ± 0.03

## Data Availability

The original contributions presented in this study are included in the article. Further inquiries can be directed at the corresponding authors.

## Supplementary Materials

The following supporting information can be downloaded at: https://www.mdpi.com/article/10.3390/microorganisms14010171/s1, Table S1: Basic physicochemical properties of tested soil. Figure S1: Bacterial physical and chemical properties test process. Figure S2: Determination of heavy metal concentrations in bacterial extracellular precipitation, surface adsorption, and intracellular accumulation. Figure S3: EC50 value of 65 UPB. Figure S4: Cd^2+^ removal of 5 UPB. Figure S5: Resistance curve of strain C7-12.

## Abbreviations

The following abbreviations are used in this manuscript:

UPB	Urease-producing bacteria
Cd	Cadmium
Pb	Lead
Cu	Copper
Zn	Zinc
SEM-EDS	Scanning Electron Microscopy–Energy Dispersive Spectrometer
FTIR	Fourier Transform infrared spectroscopy
XRD	X-ray diffraction
