# Supplementary material for: Urea-Mediated Biomineralization and Adsorption of Heavy-Metal Ions in Solution by the Urease-Producing Bacteria C7-12
Source: Microorganisms. 2026 Jan 13;14(1):171. doi: 10.3390/microorganisms14010171 (PMC12844014; doi:10.3390/microorganisms14010171)
Supplement: Supplementary file 1 [file microorganisms-14-00171-s001.zip › microorganisms-4077936-supplementary.pdf]

Table S1. Basic physicochemical properties of tested soil

Soil	pH	Organic matter (g/kg)	Total N (g/kg)	Total P (g/kg)	Total K (g/kg)	Available N (mg/kg)	Available P (mg/kg)	Available K (mg/kg)	Total Cd (mg/kg)
K	4.56±0.54	8.52±1.64	0.44±0.01	2.77±0.22	22.52±1.52	14.65±1.65	1.17±0.25	45.88±2.54	19.53±1.64
W	5.63±0.66	19.67±1.97	0.48±0.01	1.68±0.04	29.34±2.61	16.36±1.26	4.25±0.36	78.69±3.76	10.50±1.01
N	5.84±0.34	28.28±2.33	1.59±0.02	1.65±0.24	21.04±1.75	110.44±1.18	7.48±0.79	110.79±5.58	3.32±0.22

Note: K represents the slag solution, W denotes the contaminated soil solution, and N signifies the farmland soil solution. All subsequent instances are replaced with the corresponding letters

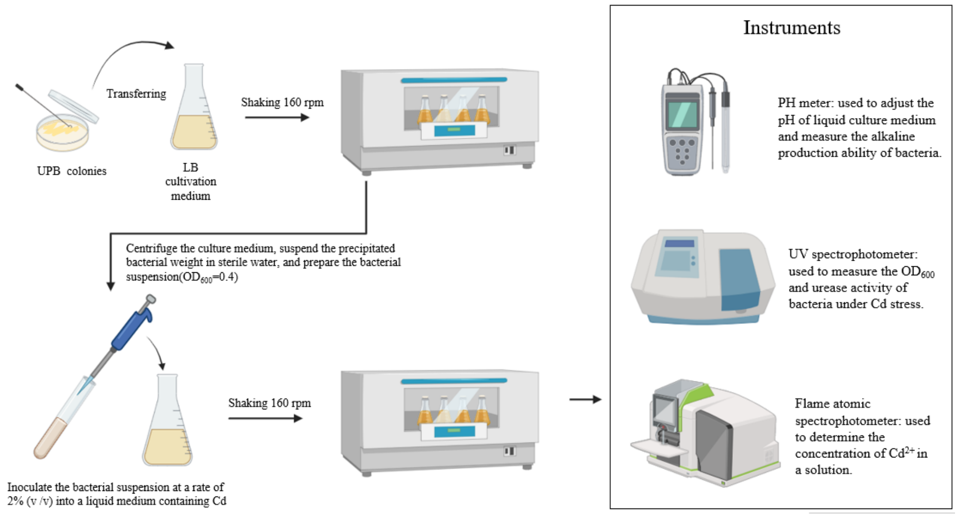

Figure S1. Bacterial physical and chemical properties test process

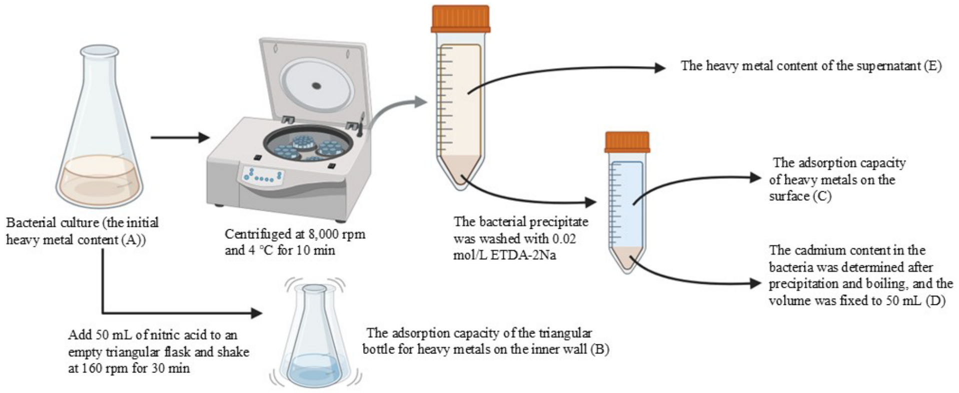

Figure S2. Determination of heavy metal concentrations in bacterial extracellular precipitation, surface adsorption, and intracellular accumulation

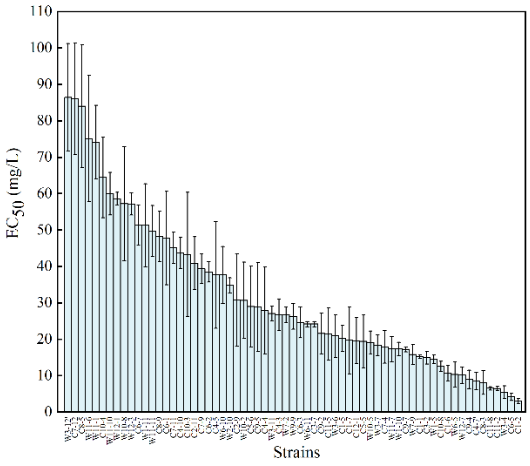

Figure S3. EC_{50} value of 65 UPB

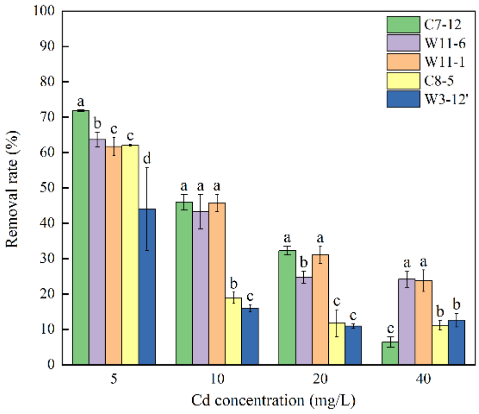

Figure S4. Cd^{2+} removal of 5 UPB

Note: The significant difference in Cd removal rate among five UPB under the same Cd concentration represented by different lowercase letters (P<0.05).

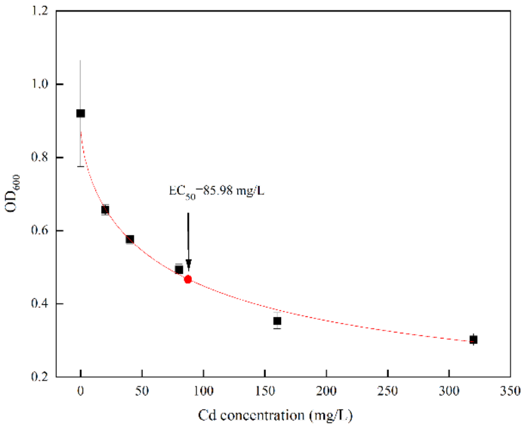

Figure S5. Resistance curve of strain C7-12
